# The design and application of an intensive care unit point-of-care nursing handover checklist based on the situation, background, assessment, and recommendation technique

**DOI:** 10.3389/fpubh.2022.1029573

**Published:** 2022-11-22

**Authors:** Lei Wang, Yu-Jie Ma, Xiao-Ting Chen, Jie Zhang, Tao Liu

**Affiliations:** Department of Critical Care Medicine, Air Force Medical Center, People's Liberation Army (PLA), Beijing, China

**Keywords:** critical patients, SBAR model, checklist, ICU, nursing, handover

## Abstract

**Objective:**

This study aims to analyze the effect of using an intensive care unit point-of-care nursing handover checklist based on the situation, background, assessment, and recommendation (SBAR) communication technique.

**Methods:**

An intensive care unit point-of-care nursing handover checklist was designed based on the SBAR technique, and standard point-of-care nursing handover procedures and effect assessment indicators were established to compare the occurrence of adverse handover events and nursing risks with those previously observed.

**Results:**

Before and after the application of the intensive care unit point-of-care SBAR checklist, the occurrence of missed items during the handover was 7.26 and 2.02%, inadequate preparation for handover was 28.33 and 5%, and nursing risks were 5 and 1.67%, respectively.

**Conclusion:**

Based on the SBAR technique, the application of an intensive care unit point-of-care nursing checklist reduced the occurrence of adverse handover events and nursing risks.

## Introduction

Nurses working different shifts communicate patients' treatment details and related information during the nursing handover, an important process that ensures nursing continuity. The accuracy of the nursing handover directly affects the safety and effectiveness of nursing activities (1–4). The nursing handover procedure in an intensive care unit (ICU) involves the transfer of complicated information concerning, for example, changes in patient medical conditions, various monitoring and treatment devices, catheters, treatment approaches, and the combined use of drugs, all of which must be discussed during this process (5). Although all departments have shifts, the mode is not uniform, some are oral, some are written, and there are differences in the content and the handover order. During the handover communication, the shift handover personnel often have problems such as lack of focus and omission, resulting in different quality of the shift handover. This increases treatment risks and reduces patient safety (6). The situation, background, assessment, and recommendation (SBAR) technique is a standard and structured communication method that is used to provide information comprehensively, accurately, and systematically (7–10). This technique has been widely applied in the healthcare community and has proven its advantages in the context of nursing handovers (2, 3, 11–13). However, the lack of a standard handover checklist for use in ICU nursing settings increases the risks that may arise during the handover process to some extent (14–16). SBAR mode is a good shift handover mode, which can provide comprehensive, accurate and systematic information and avoid the above shortcomings. The novelty of this study lies in the application of SBAR communication mode in ICU nursing shift handover, and the preparation of handover form template, which covers all handover contents. Nurses can combine SBAR mode with standard form to handover during shift handover, which can improve the quality of shift handover and ensure the safety of patients.

## Materials and methods

### Participants

A total of 28 full-time ICU nurses from the Department of Critical Care Medicine of Air Force Medical Center in Beijing were included in the study based on the following inclusion criteria: (1) the participant was a registered nurse; and (2) the participant had been working as an ICU nurse completing clinical activities for at least 6 months.

The exclusion criteria of the current study were as follows: (1) nurses on non-responsible shifts who did not care for patients; and (2) nurses who were engaged in ongoing education, rotation, or practice who did not work alone or hand over patients. The participants included 23 female and five male nurses.The educational backgrounds of the 28 participants were bachelor's degree (13 participants) or a junior college qualification (15 participants). The professional title of the 28 participants were “nurse-in-charge” (one participant), “senior nurse” (17 participants), or “nurse” (10 participants). The length of service of the 28 participants was < 5 years (15 participants), 5–10 years (seven participants), and >10 years (six participants), as shown in Table 1.

**Table 1:** Demographic data of the participants.

**Items**	**Data**
Gender	Female (23) male (5)
Educational background	Bachelor's degree (13) junior college qualification (15)
Professional title	Nurse-in-charge (1) senior nurse (17) nurse (10)
Length of service	< 5 years(15) 5–10 years (7) >10 years (6)

### Study design

#### Designing the ICU POC nursing handover checklist based on the SBAR technique

The head nurse of the department designed the checklist, and the design team, comprising the director, head nurse, quality control (QC) nurse, QC physician, and responsible team leader of the department, implemented it. They followed the structural framework of the standard SBAR communication model and accounted for several aspects, including the type of information, key challenges, and the items prone to be missed during ICU nursing handovers, to produce an SBAR-based ICU POC nursing handover checklist. The handover checklist included 28 items divided into four SBAR sections as follows:

- **Section 1**: The information relevant to the patient, including their bed number, name, gender, age, main diagnosis, medical condition, treatment, medication, vital signs, intake and output volume, diet, GCS, pupillary assessment, mental condition, skin, tubes, instruments, and equipment, environment, and any other relevant aspects.
- **Section 2**: The patient's background details, including medical, surgical, and allergy histories.
- **Section 3**: An assessment of the key areas for observation, potential complications, and nursing risks based on the patient's situation.
- **Section 4**: Patient recommendations, including precautions and indicators for continuous attention, based on the assessment results, as well as preventive measures in response to the nursing risks.

After the checklist was assembled, a 1-week clinical pre-trial was conducted, as well as any shortcomings were fixed to complete the final design.

#### Training for using the checklist

The group of 28 nurses received six training sessions in the form of centralized instruction, group demonstration, and clinical practice, with the head nurse as the training leader and the QC nurse as the deputy leader. The training comprised the following:

(1) **Centralized instruction**: The instruction included the basic concepts and advantages of the SBAR technique, items and methods for completing the checklist, and communication skills for handover.
(2) **Group demonstration:** The demonstration was conducted in the form of roleplay, with the responsible leader organizing the process. One nurse played the role of the nurse handing over and another as the nurse taking over, and everyone practiced at least once to increase their familiarity with the SBAR-based POC nursing handover checklist.
(3) **Clinical practice:** The POC nursing handover was conducted according to the SBAR-based ICU POC nursing handover checklist. The training leader reminded nurses about problems that may occur during the handover/takeover and followed up by explaining how to resolve these issues. To ensure the effectiveness of the training, the nurses of the responsible team were again assessed 4 weeks after completing their clinical practice. There were two parts to the assessment; the first part addressed theoretical knowledge, while the second part involved practicing shift handover and focused on the correctness and completeness of the information communicated by nurses during a shift handover, as well as the recommendations, precautions being provided, indicators requiring continued attention, and the reasonableness and effectiveness of preventive measures taken to minimize nursing risks.

#### Application of the checklist

One checklist was used for each shift. The nurse handing over completed the checklist information concerning the medical condition and treatment of the patients during the shift before the handover and conducted the handover process with the nurse taking over by providing the SBAR information. The handover and takeover nurses both confirmed and signed the checklist to complete the handover/takeover process.

The head nurse and QC nurse checked the handover/takeover processes at the start of each shift and recorded any issues that were observed. Indicators for handover quality assessment included the number of occurrences of adverse handover events and nursing risks. Adverse handover events included inadequate preparation for the handover, item(s) that were missed during the handover, unclear communication concerning medical conditions, matters specific to the specific circumstances, and preventive risk measures. Statistical comparisons were conducted using the information collected from 60 handovers before and 60 handovers after using the checklist, with a total of 1,680 handover items. The gender and age, as well as the Acute Physiology and Chronic Health Evaluation II scores of the patients before and after using the checklist, showed no significant differences.

#### Statistical analysis

This study used 28 participants to conduct a self-comparative study on the quality of shift handover before and after training. The SPSS Statistics 22.0 software program was used to analyze the obtained results. Omitted items, inadequate preparation, and nursing risks were expressed as cases or percentages (%), and the Chi-square (χ^2^) test was conducted for the comparison of the occurrence of handover events, item omission and inadequate preparation in nursing handover before and after using checklist, with *P* < 0.05 indicating statistical significance.

## Results

### Comparison of adverse handover events before and after using the checklist

The frequency of missing items was significantly different before and after using the checklist (*P* < 0.01), and the occurrence of inadequate preparation for the handover differed significantly (*P* < 0.01), as shown in Table 2. Before the application of the checklist, missing items were chiefly observed in nursing risks and preventive measures, including lung auscultation, bedside articles, medical waste, bedside card, wrist straps, and tube marking. Inadequate preparation for the handover mainly included loss or damage of bedside articles, condensate not managed, film contamination not changed, and incomplete tube marking. Following the application of the checklist, most nurses could hand over the information systematically; however, if changes in the patient's medical condition occurred alongside a protracted handover period, items could easily be missed, e.g., lung auscultation or bedside articles. The occurrence of inadequate preparation for the handover decreased when using the checklist, as shown in Tables 3, 4.

**Table 2:** Comparison of occurrence of handover events before and after using checklist [Times (%)].

**Items**	**Before**	**After**	**χ^2^**	***P*-value**
Item omitted	122 (7.26)	34 (2.02)	52.058	< 0.01
Inadequate preparation	17 (28.33%)	3 (5%)	11.760	< 0.01

**Table 3:** Comparison of item omission in nursing handover before and after using checklist [Times (%)].

**Items**	**Before**	**After**	**χ^2^**	***P*-value**
Bedside card and wrist strap	10 (16.67)	2 (3.33)	5.926	< 0.05
Standby drugs	7 (11.67)	0 (0)	7.434	< 0.01
Air bag pressure	6 (10)	0 (0)	6.316	< 0.05
Lung auscultation	22 (36.67)	11 (18.33)	4.104	< 0.05
Humidification pot of breathing machine	14 (23.33)	5 (8.33)	5.065	< 0.05
Tube marking	8 (13.33)	2 (3.33)	3.927	< 0.05
Nursing risks and preventive measures	31 (51.67)	5 (8.33)	26.825	< 0.01
Negative pressure suction device	9 (15)	2 (3.33)	6.519	< 0.05
Bedside article and medical waste	15 (25)	7 (11.67)	4.357	< 0.05

**Table 4:** Comparison of inadequate preparation in nursing handover before and after using checklist [Times (%)].

**Items**	**Before**	**After**	**χ^2^**	***P*-value**
Condensate not handled	9 (15)	1 (1.67)	6.982	< 0.01
Incomplete tube marking	7 (11.67)	1 (1.67)	4.821	< 0.05
Obstructed infusion pathway	1 (1.67)	0 (0)	1.008	>0.05
Contaminated film not changed	8 (13.33)	0 (0)	8.571	< 0.01
Loss or damage of bedside articles	15 (25)	1 (1.67)	14.135	< 0.01

### Comparison of nursing risks before and after using the checklist

Before the application of the SBAR-based ICU POC nursing handover checklist, the potential complications, nursing risks, and measures for preventing nursing risks were easily forgotten during the handover process, leading to outcomes that included skin damage and aspiration tube slippage. Three cases of nursing risks occurred in 60 handovers (5%). The checklist required Section 3 (“Assessment”) and Section 4 (“Patient recommendations”) to be completed during the handover. After the application of the checklist, only one case of nursing risk occurred in 60 handovers (1.67%).

## Discussion

Using an SBAR-based ICU POC nursing handover checklist for POC nursing handovers, ICU nurses could clearly understand all the relevant information. Key challenges were highlighted during the handover, and the information was handed over systematically according to the checklist, thus overcoming the problems of arbitrary conventional handover methods and ensuring the continuation of orderly nursing activities and the safety of patients. The study by Abbaszade et al. (16) showed that applying the SBAR communication technique to nursing handovers could ensure the integrity of the information communicated and improve the quality of nursing. The study by Achrekar et al. (17) concluded that the application of the standard SBAR technique in POC nursing handovers could improve communication between nurses and ensure the safety of patients. According to research by Etemadifar (18) and Herawati et al. (19), training nurses to use an SBAR-based nursing handover could improve the quality of the nursing handover and ensure the safety of patients. Martin et al. (20) conducted a literature review and concluded that using the SBAR model could improve team communication and reduce the occurrence of adverse events. McGrath et al. (21) investigated the handover process in a hemodialysis room, and the results suggested that standard SBAR-based communication could reduce errors and improve patient safety.

### Application of the ICU POC nursing handover checklist based on the SBAR technique improves the quality of the nursing handover

As shown in this study, the application of an ICU POC nursing handover checklist based on the SBAR model improved the quality of nursing handovers and reduced the occurrence of adverse handover events.

The current study showed that the application of a standard handover checklist could improve the quality of ICU nursing handovers, which was consistent with the results of the studies noted above. Possible reasons for this include the following.

(1) The POC nursing handover checklist used the SBAR model to provide comprehensive information. Using this standard checklist can help to avoid missing any details and ensure the integrity and continuity of the handover information.
(2) The checklist can serve as a reminder for nurses and support them to think clearly, thereby avoiding missing any items during the handover due to busy schedules or inadequate experience.
(3) The checklist provides traceability of the handover information and ensures continuity between shifts, thereby making it possible to effectively supervise the quality of nursing and improve the awareness of responsible nurses concerning the quality of the handover process.

### Application of the ICU POC nursing checklist based on the SBAR technique reduces the occurrence of nursing risks

In this study, the application of the ICU POC nursing checklist based on the SBAR model reduced the occurrence of nursing risks. The results of the current study were consistent with the above-noted research. Possible reasons for this include the following: (1) special training was provided regarding the use of the checklist, and the information and sequence for handovers were standardized so that the nurses taking over could quickly assimilate all the key nursing information to ensure patient safety; and (2) the content of the checklist included the assessment of nursing risks and recommendations for their prevention. Accordingly, the nurses responsible were aware of the key nursing challenges and risks and could make comprehensive judgments involving the patients and implement timely and effective preventive measures to reduce nursing risks.

Application of the SBAR-based ICU POC nursing handover checklist can help to ensure standardized systematic handovers. Moreover, it enables nursing processes to be performed according to the relevant procedures and principles and can improve the awareness of nurses about the medical conditions of their patients, thereby decreasing the occurrence of adverse events and increasing the quality of nursing care for patients in the ICU.

## Data availability statement

The original contributions presented in the study are included in the article/supplementary material, further inquiries can be directed to the corresponding authors.

## Author contributions

Conception and design of the research: LW, Y-JM, X-TC, JZ, and TL. Acquisition of data: LW, X-TC, and JZ. Analysis and interpretation of the data, statistical analysis, and writing of the manuscript: LW. Critical revision of the manuscript for intellectual content: Y-JM. All authors have read and approved the final draft.

## Conflict of interest

The authors declare that the research was conducted in the absence of any commercial or financial relationships that could be construed as a potential conflict of interest.

## Publisher's note

All claims expressed in this article are solely those of the authors and do not necessarily represent those of their affiliated organizations, or those of the publisher, the editors and the reviewers. Any product that may be evaluated in this article, or claim that may be made by its manufacturer, is not guaranteed or endorsed by the publisher.
